# Diel and tidal *p*CO_2_ × O_2_ fluctuations provide physiological refuge to early life stages of a coastal forage fish

**DOI:** 10.1038/s41598-019-53930-8

**Published:** 2019-12-03

**Authors:** Emma L. Cross, Christopher S. Murray, Hannes Baumann

**Affiliations:** University of Connecticut, Department of Marine Sciences, 1080 Shennecossett Road, 06340 Groton, CT USA

**Keywords:** Climate-change ecology, Evolutionary ecology, Marine biology, Marine biology, Climate-change ecology

## Abstract

Coastal ecosystems experience substantial natural fluctuations in *p*CO_2_ and dissolved oxygen (DO) conditions on diel, tidal, seasonal and interannual timescales. Rising carbon dioxide emissions and anthropogenic nutrient input are expected to increase these *p*CO_2_ and DO cycles in severity and duration of acidification and hypoxia. How coastal marine organisms respond to natural *p*CO_2_ × DO variability and future climate change remains largely unknown. Here, we assess the impact of static and cycling *p*CO_2_ × DO conditions of various magnitudes and frequencies on early life survival and growth of an important coastal forage fish, *Menidia menidia*. Static low DO conditions severely decreased embryo survival, larval survival, time to 50% hatch, size at hatch and post-larval growth rates. Static elevated *p*CO_2_ did not affect most response traits, however, a synergistic negative effect did occur on embryo survival under hypoxic conditions (3.0 mg L^−1^). Cycling *p*CO_2_ × DO, however, reduced these negative effects of static conditions on all response traits with the magnitude of fluctuations influencing the extent of this reduction. This indicates that fluctuations in *p*CO_2_ and DO may benefit coastal organisms by providing periodic physiological refuge from stressful conditions, which could promote species adaptability to climate change.

## Introduction

Rising anthropogenic carbon dioxide emissions are acidifying and warming our oceans at an unprecedented rate^1–3^. Current understanding of biological responses to ocean acidification is largely based on experimental exposures to static conditions that are projected to occur over centuries in the average surface ocean (400–2,200 μatm)^3^. Most marine species, however, spend all or parts of their life in coastal environments^4^, where upwelling, riverine input, nutrient loading and higher biological productivity cause generally higher and more variable *p*CO_2_ levels^5–10^. In addition, nutrient pollution increasing primary production and microbial respiration often exacerbates acidification and loss of dissolved oxygen (DO) in coastal habitats^11,12^. Hence, upwelling regions already periodically experience 2,200 μatm^13^ while some nearshore coastal habitats (e.g. saltmarshes and mangrove lagoons) can temporarily reach 4,500 μatm due to diel fluctuations in community metabolism^14^. These *p*CO_2_ and DO fluctuations occur on tidal, diel, seasonal and interannual time scales^15^. In well mixed coastal zones, tidal and diel fluctuations are primarily driven by changes in net ecosystem metabolism from net autotrophy during the day (low *p*CO_2_, high DO), to net heterotrophy during the night (high *p*CO_2_, low DO)^12,14,16^. Seasonal fluctuations in temperature and stratification often elevate *p*CO_2_ and decrease DO conditions during the biologically most productive summer months. As temperatures decrease, respiration rates decline and stratification is disrupted, causing *p*CO_2_ to decrease and DO levels to rise again. Under future climate change, these *p*CO_2_ × DO cycles are expected to increase in severity and duration of extreme conditions as absorption of atmospheric CO_2_ will reduce seawater buffering capacity while elevated temperatures will increase microbial respiration of organic matter^8,12,17,18^.

While organismal responses to hypoxia have been studied for decades revealing negative direct effects on survival, growth, physiology, behaviour and distributions of marine fish^19–22^, potential impacts of ocean acidification on marine organisms have only in recent decades received increasing attention^23,24^. Similarly to hypoxia, negative eco-physiological and behavioural responses to projected *p*CO_2_ levels have been documented for a wide range of marine fish species^25^. However, combined impacts of acidification and hypoxia remain understudied, particularly with respect to *p*CO_2_ and DO fluctuations^12^. Initial research on marine fish suggested that low oxygen impacts dominate over elevated *p*CO_2_^26–29^, while others demonstrated more severe effects of the combination of acidification and hypoxia than the individual effect of each stressor^30^. Three previous studies have investigated the effects of acidification and hypoxia on the Atlantic silverside *M. menidia*^27,29,30^, an ecologically important forage fish along the east coast of North America^31,32^. This species is a valued fish model for climate sensitivity research due to its short life cycle, ease of access to wild populations and ease of experimental rearing allowing for decades of experimental expertise^32^. *M. menidia* deposits their embryos in shallow nearshore habitats^33^ which are commonly characterised by large fluctuations in *p*CO_2_ × DO levels. The Ocean Variability Hypothesis suggests that coastal species that experience large short-term *p*CO_2_ fluctuations could produce offspring that are tolerant of cycling conditions^34^. This further demonstrates the suitability of *M. menidia* as a model species for investigating the effects of fluctuating *p*CO_2_ × DO levels on coastal organisms. Early life survival and growth of *M. menidia* has previously been reported to decrease under low DO (2.5 mg L^−1^) but not low pH (pH_T_ 7.4) under static conditions^27^. This trend of greater sensitivity to low oxygen compared to elevated *p*CO_2_ was also demonstrated in mortality and surface respiration of juvenile *M. menidia* under static conditions^30^ and diel *p*CO_2_ × DO cycling^29^. Early life stages are typically most vulnerable to environmental stressors, therefore, it is paramount to determine how diel and tidal cycles of *p*CO_2_ × DO affect fish early life stages in coastal environments. Fluctuations in these stressors could be beneficial by providing temporary physiological refuge from stressful conditions, or they may be detrimental by requiring constant physiological adjustments^35,36^.

To determine how fluctuations of *p*CO_2_ and dissolved oxygen (DO) affect fish early life survival and growth, we reared *M. menidia* embryos and larvae under static and cycling *p*CO_2_ × DO treatments in four separate experiments. Treatment conditions reflect current and predicted future *p*CO_2_ and DO conditions in metabolism-driven temperate estuaries^12,14,15,17,37^. Experiments one and two quantified individual and combined effects of static high *p*CO_2_ and low DO by crossing three static *p*CO_2_ conditions (“control *p*CO_2_” – 400 μatm, “intermediate *p*CO_2_” – 2,200 μatm and “extreme *p*CO_2_” – 4,500 μatm) with four static DO conditions (“normoxic” – 7.7 mg L^−1^, “reduced DO” – 4.0 mg L^−1^, “hypoxic” – 3.0 mg L^−1^ and “hypoxic” – 2.5 mg L^−1^; Table 1). This established our baseline understanding of multi-stressor effects on *M. menidia* early life stages. Experiments three and four assessed static, diel and tidal *p*CO_2_ × DO fluctuations of different amplitudes around three mean *p*CO_2_-DO levels (“control *p*CO_2_ – normoxic”; “intermediate *p*CO_2_ – reduced DO”; “extreme *p*CO_2_ – hypoxic”; Table 1). Three static treatments were contrasted with six cycling treatments of differing magnitudes (Table 1) and frequency (diel – 24 hours; tidal – 12 hours). All four experiments quantified five fitness-relevant early life history traits: time to 50% hatch, embryo survival, larval survival, size at hatch and larval growth rates.

**Table 1:** *p*CO_2_ × DO conditions for all static and fluctuating treatments in each mean *p*CO_2_-DO level.

Mean *p*CO_2_-DO level	Cycling Pattern	Treatment abbreviation	Experiment	*p*CO_2_ (μatm)	DO (mg L^−1^)
Control *p*CO_2_-Normoxic	Static	Control-Static	1, 2, 3, 4	387 ± 21	7.7 ± 0.2
Control *p*CO_2_-Reduced DO	Static	Control-Red	1, 2	472 ± 36	4.2 ± 0.4
Control *p*CO_2_-Hypoxic	Static	Control-Hyp	1	400 ± 1	2.6 ± 0.4
			2	520 ± 5	3.3 ± 0.7
Intermediate *p*CO_2_-Normoxic	Static	Intermediate-Norm	1, 2	2000 ± 190	7.7 ± 0.1
Intermediate *p*CO_2_-Reduced DO	Static	Intermediate-Static	1, 2, 3, 4	2309 ± 117	4.1 ± 0.4
	Small Diel Fluctuation	Intermediate-SDF	3	1166–4953	2.3–6.0
			4	876–3059	4.0–6.6
	Large Diel Fluctuation	Intermediate-LDF	3	521–9926	1.4–6.2
			4	747–8810	3.0–6.1
	Tidal Fluctuation	Intermediate-TF	3	699–8667	1.6–6.1
			4	948–9277	3.0–6.6
Intermediate *p*CO_2_-Hypoxic	Static	Intermediate-Hyp	1	2189 ± 4	2.6 ± 0.4
			2	2151 ± 19	3.1 ± 0.4
Extreme *p*CO_2_-Normoxic	Static	Extreme-Norm	1, 2	4454 ± 101	7.7 ± 0.1
Extreme *p*CO_2_-Reduced DO	Static	Extreme-Red	1, 2	4315 ± 216	4.1 ± 0.4
Extreme *p*CO_2_-Hypoxic	Static	Extreme-Static	1, 3	4681 ± 473	2.5 ± 0.4
			2, 4	4579 ± 217	3.1 ± 0.4
	Small Diel Fluctuation	Extreme-SDF	3	1872–9590	1.9–4.2
			4	2341–6490	3.0–5.2
	Large Diel Fluctuation	Extreme-LDF	3	1058–15970	1.1–5.3
			4	1258–12624	2.6–5.7
	Tidal Fluctuation	Extreme-TF	3	1349–17013	1.6–5.0
			4	1515–11658	2.7–5.9

Values are mean ± S.D. for static treatments or ranges for fluctuating treatments.

## Results

### Experiments one and two: static pH × DO experiments

**Figure 1:**
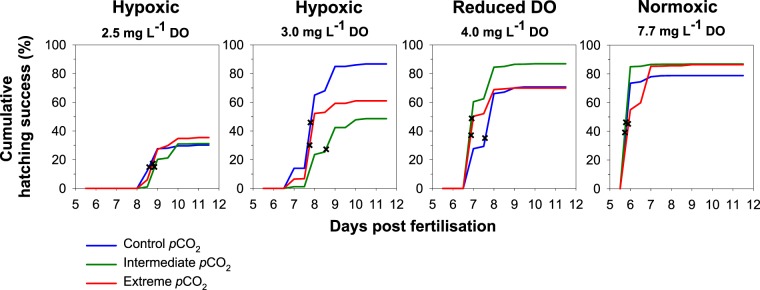
Cumulative hatching success (%) of *M. menidia* offspring reared at three static *p*CO_2_ levels (see legend) crossed with four DO concentrations (L-R: 2.5 mg L^−1^, 3.0 mg L^−1^, 4.0 mg L^−1^ and 7.5 mg L^−1^). Lines represent treatment means pooled from both experiments. Crosses indicate 50% of larvae hatched in treatment.

**Figure 2:**
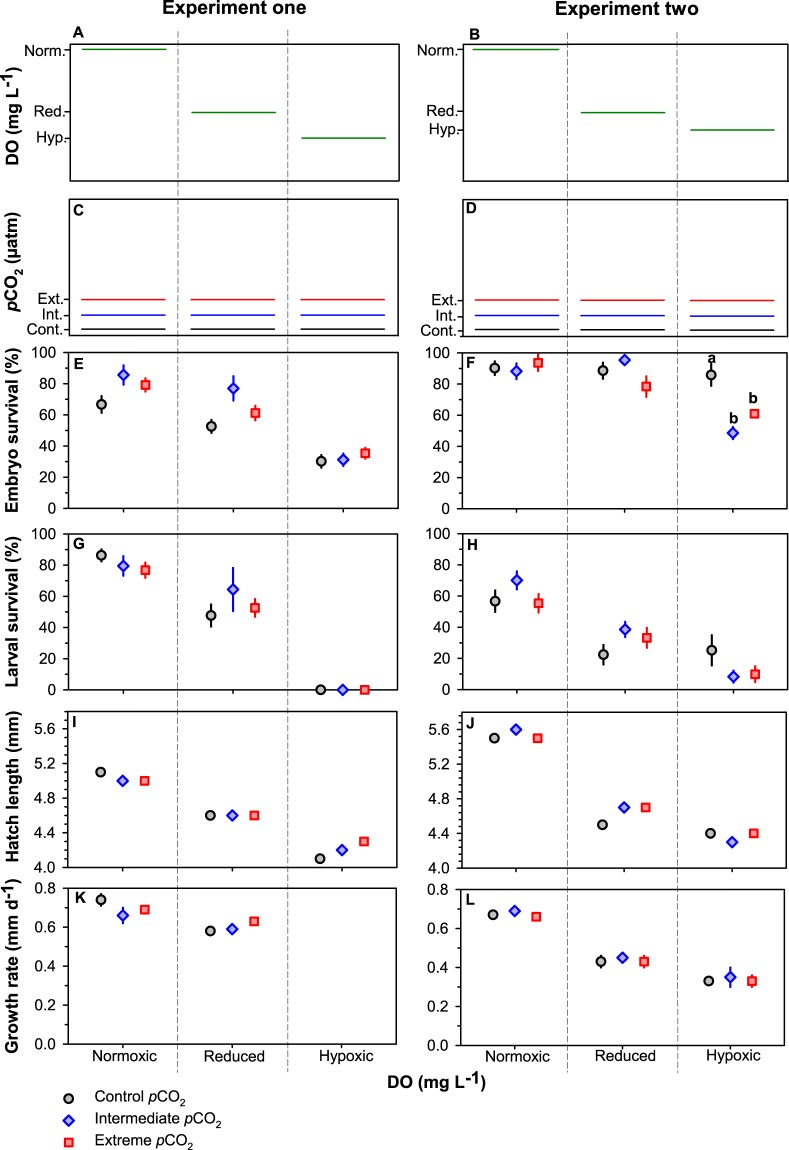
*Effects of static pCO*_2_ *×* *DO conditions on survival and growth of early life M. menidia*. Schematics of static DO (normoxic, reduced DO and hypoxic; **A**,**B**) and *p*CO_2_ (control, intermediate and extreme; **C**,**D**) conditions from nine treatments in each experiment. Embryo survival (%; **E**,**F**), larval survival (%; **G**,**H**), hatch length (mm; **I**,**J**) and growth rate (mm day^−1^; **K**,**L**) from *M. menidia* offspring reared under three static *p*CO_2_ levels crossed with four static DO concentrations from experiments one (left column) and two (right column). Values are treatment means ± S.E. Different lowercase letters represent significant interactions of *p*CO_2_ and DO conditions.

Time to 50% hatch was shortest under normoxic conditions with 50% of larvae hatched after 6 days post fertilisation (dpf). Hatching was delayed to 7 dpf in 4.0 mg L^−1^, 8 dpf in 3.0 mg L^−1^ and 9 dpf in 2.5 mg L^−1^ (Fig. 1). Elevated *p*CO_2_ did not impact hatch timing. Declining DO significantly reduced embryo survival (Linear mixed effects model, χ^2^ = 84.79, p < 0.001; Fig. 2E,F). Declining DO conditions decreased embryo survival from 84 ± 3% S.E. in 7.7 mg L^−1^ to 74 ± 4% S.E. in 4.0 mg L^−1^, 65 ± 5% S.E. in 3.0 mg L^−1^ and 32 ± 2% S.E. in 2.5 mg L^−1^ across experiments. Elevated *p*CO_2_ levels only impacted embryo survival at 3.0 mg L^−1^ with a 37% decrease at 2,200 μatm (Tukey, p < 0.001) and a 19% decrease at 4,500 μatm (Tukey, p = 0.006; Fig. 2F) relative to 400 μatm. Larval survival, size of newly hatched larvae and post-hatch growth rates decreased with declining DO but were statistically unaffected by *p*CO_2_ (Table 2; Table S1, Fig. 2G–L).

**Table 2:** Overview of static and fluctuating *p*CO_2_ × DO effects on *M. menidia* offspring survival and growth. Green symbols = experiments one & two; purple symbols = experiment three; orange symbols = experiment four. Crosses = statistically unaffected response trait; arrows = increase/decrease in response trait.

Response traits	Experiments one & two: static *p*CO_2_ × DO experiments			Experiments three & four: fluctuating *p*CO_2_ × DO experiments		
	Increased *p*CO_2_ effect	Decreased DO effect	Increased *p*CO_2_ × decreased DO effect	Static mean *p*CO_2_-DO level effect	Fluctuating *p*CO_2_ × DO effect	
					Intermediate level	Extreme level
Time to 50% hatch						
Embryo survival						
Larval survival						
Size at hatch						
Post-hatch growth rates						

### Experiments three and four: Fluctuating *p*CO_2_ × DO experiments

#### Experiment three

**Figure 3:**
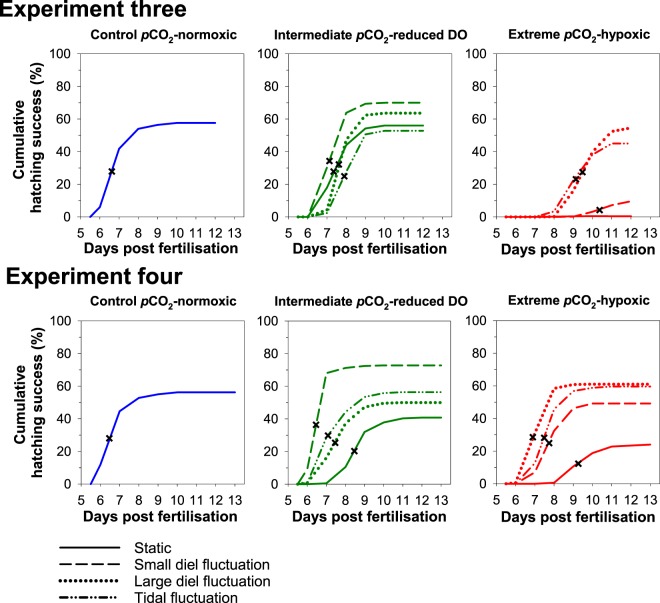
Cumulative hatch success (%) of *M. menidia* offspring reared in static and fluctuating *p*CO_2_ × DO conditions (see legend) under control *p*CO_2_–normoxic (left column), intermediate *p*CO_2_–reduced DO (middle column) and extreme *p*CO_2_–hypoxic levels (right column) across experiment three (top row) and experiment four (bottom row). Lines represent treatment mean and crosses indicate 50% of larvae hatched in treatment.

**Figure 4:**
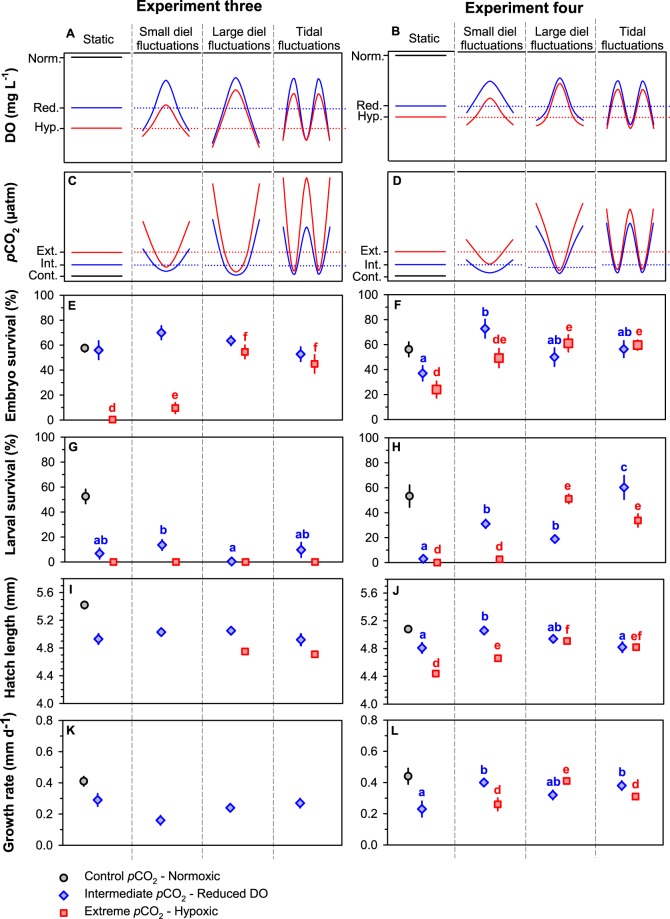
*Effects of static vs. fluctuating pCO*_2_ × *DO conditions on survival and growth of early life M. menidia*. Schematics of DO (**A**,**B**) and *p*CO_2_ (**C**,**D**) conditions over a 24 hour period from three static and six cycling treatments of different magnitudes and frequencies (small diel fluctuation, large diel fluctuation, tidal fluctuation) in the three mean *p*CO_2_-DO levels (see legend). Embryo survival (%; **E**,**F**), larval survival (%; **G**,**H**), hatch length (mm; **I**,**J**) and growth rate (mm day^−1^; **K**,**L**) from *M. menidia* offspring reared in different *p*CO_2_ × DO cycling patterns from experiment three (left column) and four (right column). Values are treatment means (±S.E.). Different lowercase letters represent significant differences between cycling patterns within the intermediate *p*CO_2_–reduced DO level (a–c; blue diamonds) and within the extreme *p*CO_2_–hypoxic level (d–f; red squares).

Time to 50% hatch was shortest in control conditions with 50% of larvae hatched by 6 dpf. Hatching was delayed to 7 dpf in all intermediate *p*CO_2_-reduced DO treatments with cycling pattern having no effect (Fig. 3). No hatching occurred in the extreme-static treatment, however, hatching was delayed to 9 dpf in the extreme-LDF and extreme-TF and further delayed to 10 dpf in the extreme-SDF treatment (Fig. 3). In the static treatments, embryo survival was significantly reduced in extreme *p*CO_2_-hypoxic conditions compared to the intermediate *p*CO_2_-reduced DO and control *p*CO_2_-normoxic conditions (Linear model, F_2,11_ = 212.77, p < 0.001; Fig. 4E). Cycling treatments increased embryo survival in the extreme *p*CO_2_-hypoxic level with the highest survival occurring in the extreme-LDF (Fig. 4E; Table S2; Tukey, p < 0.001). Cycling pattern in the intermediate *p*CO_2_-reduced DO level, however, did not affect embryo survival (Tukey, p = 0.270). Similarly, larval survival, mean size of newly hatched larvae and post-hatch growth rates all decreased with increasing *p*CO_2_ and declining DO conditions in the static treatments (Table S2) with cycling pattern having no effect (Table 2; Table S3; Fig. 4G,I,K). Larval survival was low (<14%) or 0% in all intermediate *p*CO_2_-reduced DO and extreme *p*CO_2_-hypoxic treatments, respectively, after only 6 days post hatch (dph) and embryo survival was too low (<10%) to obtain size at hatch measurements in the extreme-static and extreme-SDF treatments. Complete larval mortality precluded estimation of growth rates for all extreme *p*CO_2_-hypoxic treatments. These trends were probably due to the daily minimum DO values in all intermediate *p*CO_2_-reduced DO and extreme *p*CO_2_-hypoxic treatments being below the oxygen tolerance limit of *M. menidia* (<3.0 mg L^−1^; Table 1).

#### Experiment four

In the static treatments, time to 50% hatch was shortest under control conditions with 50% of larvae hatched by 6 dpf, which was delayed in intermediate *p*CO_2_-reduced DO conditions to 8 dpf and further delayed to 9 dpf in extreme *p*CO_2_-hypoxic conditions (Fig. 3). Cycling treatments, however, shortened time to 50% hatched in both levels. In the intermediate *p*CO_2_-reduced DO level, time to 50% hatch was shortened to 7 dpf in the LDF and TF and further shortened to 6 dpf in the SDF treatment (Fig. 3). In the extreme *p*CO_2_-hypoxic level, time to 50% hatch was shortened to 7 dpf in the SDF and TF and further shortened to 6 dpf in the LDF treatment (Fig. 3). In the static treatments, all response traits decreased with increasing *p*CO_2_ and declining DO conditions (Table 2; Table S3) with all cycling treatments alleviating these negative effects (Tables 2 and S4; Fig. 4F,H,J,L). In the intermediate *p*CO_2_-reduced DO level, highest embryo survival, largest mean hatch length and fastest growth rates occurred in the SDF treatment. In the extreme *p*CO_2_-hypoxic level, however, all response traits were highest in the LDF. There was also no significant difference between LDF and TF in the majority of response traits in both levels (Fig. 4F,H,J,L).

## Discussion

By simulating both static and fluctuating *p*CO_2_ × DO environments, this study advanced our previous understanding on how both stressors affect early life stages^27^ of an ecologically important forage fish and model in climate sensitivity research^32^. While our static 3 × 3 designs (experiments one and two) improved quantification of baseline reaction norms over previous 2 × 2 approaches^27^, our findings for fluctuating conditions – as they naturally occur in nearshore habitats – shed new light on whether such fluctuations are detrimental or beneficial to marine organisms. First, when exposed to static *p*CO_2_ × DO conditions, survival and growth of early life *M. menidia* were more sensitive to reduced DO than elevated *p*CO_2_. Both embryo and larval survival severely declined with decreasing DO and resulted in complete offspring mortality at 2.5 mg L^−1^. However, even DO levels of 4.0 mg L^−1^, which are above the operational hypoxia threshold and periodically already occur in productive nearshore habitats^14,15^, significantly reduced offspring survival in this species. Declining DO levels also delayed hatching, reduced hatch size and post-hatch growth rates. In contrast, elevated *p*CO_2_ did not affect most response traits under reduced DO and normoxic conditions. At 3.0 mg L^−1^ DO, however, embryo survival decreased by 33% at 2,200 μatm and by 15% under 4,500 μatm relative to controls. These findings in *M. menidia* ealry life stages are similar to previous studies that documented negative *p*CO_2_ × DO survival effects in *M. menidia* larvae and juveniles^27,30^. Low DO and high *p*CO_2_ may elicit fatal effects in this species, possibly due to decreased functional capacity of pH-sensitive tissues and/or additional metabolic costs for acid-base regulation^12,38–40^, however, this warrants further investigation.

The second part of this study demonstrated that diel and tidal *p*CO_2_ × DO fluctuations reduced the negative survival and growth responses observed under static *p*CO_2_/DO conditions. At each mean *p*CO_2_-DO level, higher embryo and larval survival, shorter time to 50% hatch, larger size at hatch and faster post-hatch growth rates occurred in all cycling treatments relative to the static treatment. Fluctuating conditions therefore comprised a physiological refuge to early life *M. menidia* allowing temporary recovery from detrimental *p*CO_2_ and DO levels when conditions oscillated to more favourable conditions^36^. A recent review on the direct impacts of *p*CO_2_ variability on biological responses revealed that out of 24 observations (eight published papers^26,28,29,41–45^) on fish survival, growth, respiration, behaviour and otolith development^46^, five were positive, one negative and 18 were neutral. Consistent with our study, diel *p*CO_2_ fluctuations reduced negative impacts of static *p*CO_2_ conditions on larval growth in pink salmon, *Oncorhynchus gorbuscha*^47^ and on juvenile growth and behavioural responses in coral reef fishes *Acanthochromis polyacanthus* and *Amphiprion percula*^42,44^. Static elevated *p*CO_2_ conditions can alter aerobic capacity in some fish^25,47,48^ possibly due to increased metabolic costs regulating acid-base balance^49–51^. A diel *p*CO_2_ cycling environment could be less energetically expensive than static elevated *p*CO_2_ environments as the cost of acid-base regulation decreases during more favourable conditions in the diel cycle. Aerobic scope is thus increased for other fitness-relevant traits such as growth and other physiological mechanisms^44^. Neutral survival, growth and/or behavioural responses to *p*CO_2_/DO cycling have occurred in juvenile estuarine weakfish *Cynoscion regalis*^28^ and in juvenile striped killifish *Fundulus majalis*, mummichog *Fundulus heteroclitus*, striped bass *Morone saxatilis* and the Atlantic silverside *M. menidia*^29^. Reported negative responses to large diel cycling in pH, which varies with *p*CO_2_, and DO (pH 6.80–8.10, 1.0–11.0 mg L^−1^ DO) include decreased growth after 10 days exposure in juvenile summer flounder *Paralichthys dentatus* with >90% mortality occurring after 2–3 weeks exposure^26^. These trends demonstrate species-specific responses to fluctuating *p*CO_2_ × DO conditions with some species capable of maintaining physiological homeostasis whereas others require constant physiological adjustments to changing environmental conditions leading to detrimental impacts.

The degree to which *p*CO_2_/DO fluctuations ameliorated the negative effects observed under static conditions depended on the magnitude but not on the frequency of these fluctuations. At the intermediate *p*CO_2_-reduced DO level, small diel fluctuations best improved all response traits, potentially because offspring did not experience the most extreme *p*CO_2_-hypoxic conditions (daily min. 4.0 mg L^−1^, daily max. 3,059 μatm; Table 1) that temporarily occurred in the two other cycling treatments (LDF daily min. 3.0 mg L^−1^, daily max. 8,810 μatm; TF daily min.: 3.2 mg L^−1^, 9,277 μatm; Table 1). The *p*CO_2_/DO conditions in the intermediate-SDF are also the most similar to late spring/early summer conditions in the Atlantic silverside spawning habitat^14^. Similarly, small diel *p*CO_2_ fluctuations (1,000 ± 300 μatm), that typically occur in the coral reef fish habitats best ameliorated the negative survival and growth effects observed under static *p*CO_2_ conditions in *Acanthochromis polyacanthus*^44^. In contrast, large diel fluctuations at the extreme *p*CO_2_-hypoxic level had the greatest reduction in the negative effects of static *p*CO_2_ × DO conditions on all response traits. These offspring experienced more optimal *p*CO_2_ × DO conditions (<2,000 μatm, >5.2 mg L^−1^; Table 1) for short time periods every 24 hours whereas offspring reared in the extreme-SDF treatment were constrained to higher *p*CO_2_ and lower DO levels (2,341–6,490 μatm, 3.0–5.2 mg L^−1^; Table 1). All response traits also did not differ between large diel fluctuations and tidal fluctuations in both mean *p*CO_2_-DO levels demonstrating that magnitude of fluctuations influenced biological responses more than the frequency of oscillations. Duration of exposure to hypoxic DO conditions (<3.0 mg L^−1^) also influenced the severity of negative impacts on all response traits. In experiment three, complete larval mortality occurred in all extreme *p*CO_2_-hypoxic treatments after only 6 days post hatch, whereas in experiment four 38% and 51% of larvae survived to 10 dph in extreme-LDF and extreme-TF treatments, respectively, likely because minimum DO levels were increased by 1.0 mg L^−1^. This suggests that even though average DO levels in the extreme-LDF and extreme-TF treatments were around 6.0 mg L^−1^, the occurrence of DO conditions below 3.0 mg L^−1^ for 10 hours per day (experiment three) compared to only 3 hours in experiment four, proved fatal for Atlantic silverside offspring.

Embryos appeared to be more resilient to low DO conditions under static and fluctuating regimes than larvae. Acclimation to hypoxia in embryos most likely occurs through reducing their oxygen requirements by depressing metabolic rates^52^. Decreased size at hatch with declining static DO conditions indicated embryonic metabolic depression. Under fluctuating conditions, metabolic rates most likely increased as their environment oscillated to more optimal DO conditions, elevating oxygen uptake and producing a larger size at hatch relative to their static treatment. Atlantic silverside embryos attach to benthic vegetation in shallow coastal environments^33^, therefore, this apparent hypoxia tolerance may be an adaptation to periodic hypoxia that typically occurs in the summer months in their spawning habitat. Lower larval survival, however, suggests that this next life stage cannot depress metabolism to counteract declining oxygen supply once feeding and swimming commences. We observed that larvae in static low DO conditions and in fluctuating treatments during periods of DO levels below 4.0 mg L^−1^ were constrained to the immediate surface waters where higher DO conditions persist at the air-water interface. Aquatic surface respiration is a compensatory behaviour exhibited by some fishes to hypoxia, which has previously been reported in *M. menidia* juveniles under extreme static low DO conditions^30^ and larvae reared in fluctuating conditions when DO is <1.6 mg L^−1 ^^29^. In nature, this behaviour likely increases predation risk and reduces foraging ability of developing larvae^29^.

This study confirmed *M. menidia* offspring survival and growth to be more sensitive to reduced DO than elevated *p*CO_2_ under static treatments. Diel and tidal cycling of *p*CO_2_/DO, however, ameliorated these negative effects of static elevated *p*CO_2_ and decreased DO conditions. Furthermore, the extent of alleviation was influenced by the mean *p*CO_2_-DO level and the magnitude of fluctuation. To date, most ocean acidification experiments have been conducted using static elevated *p*CO_2_ conditions based on open ocean projections, however, shallow coastal environments experience substantial fluctuations in *p*CO_2_ as well as DO on tidal, daily, seasonal and interannual time scales^14,15^. Here we utilised a computer-controlled *p*CO_2_/DO-manipulation system to alter *p*CO_2_ and DO conditions every hour to incrementally increase or decrease *p*CO_2_ and DO on varying magnitudes and frequencies around an intermediate mean *p*CO_2_-DO level, mimicking common conditions during late spring and summer in coastal systems, and an extreme *p*CO_2_-DO level, simulating potential future conditions during late spring and summer with increased eutrophication and climate change. This revealed that fluctuating *p*CO_2_/DO conditions provide physiological refuge to *M. menidia* early life stages indicating that the effects of future acidification and hypoxia may be less severe than experiments using static *p*CO_2_/DO conditions have implied^27,29,30^. This is consistent with the Ocean Variability Hypothesis that suggests the most *p*CO_2_ tolerant marine organisms are those that experience large short-term *p*CO_2_ fluctuations in their natural environment^34^. Fluctuating *p*CO_2_ × DO environments could promote species adaptability to long-term change, therefore, incorporating natural *p*CO_2_/DO variability to multi-stressor experiments is crucial to more accurately assess the effects of anthropogenic change on coastal marine organisms.

## Material and Methods

### Specimen collection and fertilisation

Wild, spawning ripe Atlantic silversides were collected on four occasions in late spring and early summer in 2017 (8^th^ May and 8^th^ June) and 2018 (14^th^ May and 14^th^ June) from Mumford Cove (41° 32′27′ N, 72° 1.59′ W), a shallow embayment in Long Island Sound, Connecticut, USA. Adults were sampled using a 30 × 2 m beach seine with a 3 mm mesh size at high tide during new moon or full moon due to the semilunar spawning periodicity of *M. menidia*. For each experiment, 12–28 females were strip spawned with their eggs evenly distributed onto 1 mm mesh size window screens submerged in seawater. Milt from 23–39 males was mixed with 500 mL of seawater, carefully poured into the spawning dishes and left to fertilise the eggs for 30 minutes. Chorionic filaments in the embryos uncoil once fertilised and attach to the window screen, allowing accurate enumeration of 100 fertilised embryos into each replicate rearing container within 2 hours of fertilisation. Numbers and total lengths of spawners used in each experiment are in Table S4. This standardised strip-spawning protocol allowed the random distribution of embryos across treatments and maximised fertilisation success^41,53–55^.

### Experimental design

All four experiments were separately conducted in a computer-controlled *p*CO_2_/DO-manipulation system composed of nine individual recirculation units as detailed elsewhere^53^. UV-sterilised and 1μm-filtered natural seawater was used in each recirculating unit, which consisted of a 40L header tank, a 240L experimental tank and a 90L sump tank. Five replicate 20L rearing containers were used in each experimental tank. Every hour, pH and DO levels were manipulated using LabView (National Instruments) software to control sampling pumps and water solenoids for each recirculating unit to sequentially pump seawater for 7 minutes past a central pH electrode (Hach pHD Digital Electrode calibrated twice weekly using NIST 2-point pH buffers) and an optical dissolved oxygen (DO) probe (Hach LDO Model 2). Measured pH and DO conditions were then compared to pre-determined levels for each hour and adjusted by injecting 100% bone dry CO_2_ gas (AirGas) into the header tank, nitrogen gas (AirGas) into the sump or CO_2_-stripped air into the sump via different gas solenoids. Temperature was maintained by thermostats (Aqualogic) connected to submersible heaters and chillers (DeltaStar). Optimal temperatures (24 °C), light conditions (15 h light: 9 h dark) and salinity (30–33 psu) persisted throughout experiments^31^. LabView logged measured and set pH, DO and temperature conditions hourly for each recirculation unit. Daily checks of salinity (Refractometer) and water quality (Saltwater Ammonia Test Kit, API, <0.25 ppm) were conducted and maintained through daily waste siphoning and 25% water changes.

To determine the carbonate chemistry of each treatment, seawater samples were collected in 300 mL borosilicate bottles by siphoning seawater from each experimental tank through a 10 μm filter at three time points throughout each experiment. These samples were stored in 4 °C with total alkalinity (*A*_*T*_) later measured via endpoint titration (Mettler Toledo G20 Potentiometric Titrator). Accuracy of measurements (±1%) was verified with certified reference material for *A*_*T*_ in seawater (Dr. Andrew Dickson, University of California San Diego, Scripps Institution of Oceanography). Partial pressure of CO_2_ (*p*CO_2_, μatm) was calculated using CO2SYS (https://cdiac.ess-dive.lbl.gov/ftp/co2sys/) based on measured daily average, minimum and maximum pH_NIST_ and experiment averages of *A*_*T*_, temperature and salinity using K1 and K2 constants^56–58^ (Tables S5, S6 and S7).

### Experimental *p*CO_2_ and DO conditions

Treatment conditions reflect current and predicted future *p*CO_2_ and DO conditions in metabolism-driven temperate estuaries^12,14,15,17,37^. The *p*CO_2_ target for the control treatment was 400 μatm corresponding to current conditions in coastal habitats before the onset of biological production in spring. The target for the intermediate level was 2,200 μatm, resembling common conditions during late spring and summer in coastal systems and also an important benchmark in ocean acidification research as the maximum prediction within the next 300 years^1^. The *p*CO_2_ target for the extreme treatment is 4,500 μatm, which although currently uncommon in coastal habitats, it represents potential future conditions during late spring and summer with increased eutrophication and climate change^17,37^. The target DO levels were determined from long-term monitoring of co-varying pH and DO variations in coastal systems^14^ and set as 7.5 mg L^−1^ (normoxic, ~100% saturation), 4.0 mg L^−1^ (reduced DO, ~ 55% saturation) and 2.5 mg L^−1^ (hypoxic, ~33% saturation, experiment one), respectively. The extreme DO level in experiment two was raised to 3.0 mg L^−1^ (hypoxic, ~42% saturation) to avoid complete mortality exhibited in experiment one. Fish in experiments one and two were reared in these conditions as static levels using a full factorial 3 pH × 3 DO design (Fig. 2A–D). Fish in experiment three were reared in these conditions as target means with levels fluctuating with different amplitudes (Table S6) and two different frequencies (diel – 24 hours, tidal – 12 hours; Fig. 4A–D). In experiment four, the daily maximum *p*CO_2_ were decreased by 2,000 μatm and the daily minimum DO levels were increased by 1.0 mg L^−1^, therefore, slightly reducing the three different amplitudes (Table S7) to avoid complete larval mortality observed in the extreme level in experiment three.

### Response traits

Five response traits were measured to determine the effects of static and fluctuating *p*CO_2_ and DO conditions on the survival and growth of early life stages of the Atlantic silverside. After 5 days post fertilisation (dpf), embryos were checked every 12 hours for hatched larvae, which were counted and moved from the embryo baskets to the main rearing container. Time to 50% hatch was determined as the number of days until 50% of the total larvae hatched in each treatment since the day of fertilisation. Embryo survival (%) was quantified as the total number of one-day post-hatch larvae divided by the initial number of 100 embryos. To measure hatch length (total length, TL, ±0.01 mm), a random sub-sample of 10 larvae on the first day of hatching were preserved in 5% formaldehyde in freshwater solution buffered with saturated sodium tetraborate and later measured using Image Pro Premier (V9.0, Media Cybernetics). Newly hatched larvae were provided with equal rations of powdered weaning diet (Otohime Marine Fish Diet, size A1, Reed Mariculture) to stimulate feeding. Larvae were also fed daily with *ad libitum* rations of newly hatched brine shrimp nauplii (*Artemia salina*, brineshrimpdirect.com). Larval survival (%) was quantified as the number of survivors at 10 or 15 dph divided by the number of survivors at hatch minus 10 initial sub-samples. To calculate growth rate, final TL of all survivors at the end of the experiment was measured using Image Pro and the following equation:

$$Growth\,rate=\frac{mean\,final\,TL\mbox{--}mean\,hatch\,TL}{number\,of\,days\,reared\,posthatch}$$

### Statistical analysis

For experiments one and two, linear mixed effects models were conducted to determine significant effects of static *p*CO_2_, static DO or their interaction (fixed factors) and experiment (random factor) for each response trait using the following model:

$${Re}sponse\,trait=pC{O}_{2}+DO+pC{O}_{2}\,x\,DO+\exp eriment+error$$

Post-hoc Tukey tests were used for pairwise comparisons. For response traits exhibiting significant differences between experiments, further linear models using only the fixed factors and Tukey tests were conducted for each experiment.

As experiments three and four were not fully crossed (no fluctuating treatments around control conditions), linear models were first used to determine significant differences of mean *p*CO_2_-DO level (control *p*CO_2_-normoxic, intermediate *p*CO_2_-reduced DO or extreme *p*CO_2_-hypoxic) of only static treatments on each response trait. Further linear models were then conducted to determine significant differences of mean *p*CO_2_-DO level (intermediate *p*CO_2_-reduced DO or extreme *p*CO_2_-hypoxic), cycling pattern (static, small diel fluctuation, large diel fluctuation or tidal fluctuation), or their interaction for each response trait using the following model:

$${Re}sponse\,trait=level+cycling\,pattern+level\,x\,cycling\,pattern+error$$

Post-hoc Tukey tests were performed when significant differences were identified. Residuals of all models were checked for variance homogeneity and normality using Levene’s and Shapiro-Wilk tests (p < 0.05), respectively. No statistics were performed on the time to 50% hatch data. Statistical analyses were computed using RStudio^59^ with the *lme4* package^60^ for linear mixed effects models and the *emmeans* package^61^ for the post-hoc Tukey tests.

### Ethics

Institutional Animal Care and Use Committee (IACUC) guidelines on fish husbandry were used and all experiments were approved by IACUC of the University of Connecticut (no. A14-032, A17-043).

## Supplementary information

Supplementary Information

## Data Availability

Datasets are publicly available from the BCO-DMO data portal via the following DOIs: survival dataset - 10.1575/1912/bco-dmo.777117.1, growth dataset - 10.1575/1912/bco-dmo.777130.1 and carbonate chemistry dataset - 10.1575/1912/bco-dmo.777144.1.
