# Supplementary material for: Diel and tidal pCO2 × O2 fluctuations provide physiological refuge to early life stages of a coastal forage fish
Source: Sci Rep. 2019 Dec 3;9:18146. doi: 10.1038/s41598-019-53930-8 (PMC6890771; doi:10.1038/s41598-019-53930-8)
Supplement: Supplementary file 1 — Supplementary Information [file 41598_2019_53930_MOESM1_ESM.pdf]

Supplementary Information

Diel and tidal pCO_2 x DO fluctuations provide physiological refuge to early life stages of a coastal forage fish

Emma L. Cross, Christopher S. Murray, Hannes Baumann

Table S1: *Experiments one and two* - Statistical results from linear mixed effects models determining if static pCO_2, static DO or the interaction of these two factors significantly impacted survival and growth of *M. menidia* early life stages. *p* values in bold denote significant differences.

**Response trait**	**Fixed effects**	**$\chi 2$**	**d.f.**	***p* value**
Embryo survival	pCO_2	8.20	6	0.182
	DO	84.79	6	**<0.001**
	pCO_2 x DO	19.84	4	**<0.001**
Larval survival	pCO_2	8.90	6	0.179
	DO	123.48	6	**<0.001**
	pCO_2x DO	7.05	4	0.133
Hatch length	pCO_2	8.00	6	0.238
	DO	191.98	6	**<0.001**
	pCO_2 x DO	5.77	4	0.217
Growth rate	pCO_2	2.80	6	0.833
	DO	99.67	6	**<0.001**
	pCO_2 x DO	2.79	4	0.593

Table S2: *Experiments three and four* - Statistical results from linear models determining if mean pCO_2-DO level (control pCO_2-normoxic, intermediate pCO_2-reduced DO, extreme pCO_2-hypoxic) significantly impacted survival and growth of *M. menidia* early life stages in static treatments only. *p* values in bold denote significant differences.

Experiment	Response trait	F	d.f.	*p* value
Three	Embryo survival	212.77	2, 11	**<0.001**
	Larval survival	49.91	2, 11	**<0.001**
	Hatch length	31.56	1, 7	**0.001**
	Growth rate	6.34	1, 6	**0.045**
Four	Embryo survival	6.87	2, 11	**0.012**
	Larval survival	50.90	2, 12	**<0.001**
	Hatch length	40.23	2, 8	**<0.001**
	Growth rate	9.76	1, 6	**0.026**

Table S3: *Experiments three and four* - Statistical results from linear models determining if mean pCO_2-DO level (intermediate pCO_2-reduced DO or extreme pCO_2-hypoxic), cycling pattern (static, small diel fluctuation, large diel fluctuation or tidal fluctuation) or the interaction of these two factors significantly impacted survival and growth of *M. menidia* offspring. Only cycling pattern was tested in the intermediate pCO_2-reduced DO level in experiment three due to complete larval mortality in the extreme pCO_2-hypoxic treatments. *p* values in bold denote significant differences.

Experiment	Response trait	Factor	F	d.f.	*p* value
Three	Embryo survival	Level	100.587	1, 31	**<0.001**
		Cycling Pattern	27.528	3, 31	**<0.001**
		Level x Cycling Pattern	26.221	3, 31	**<0.001**
	Larval survival	Cycling Pattern	3.4726	3, 15	**0.043**
	Hatch length	Level	20.452	1, 22	**<0.001**
		Cycling Pattern	1.418	3, 22	0.264
		Level x Cycling Pattern	0.655	1, 22	0.427
	Growth rate	Cycling Pattern	2.887	3, 7	0.112
Four	Embryo survival	Level	1.393	1, 31	0.247
		Cycling Pattern	8.245	3, 31	**<0.001**
		Level x Cycling Pattern	5.769	3, 31	**0.038**
	Larval survival	Level	18.924	1, 32	**<0.001**
		Cycling Pattern	71.562	3, 32	**<0.001**
		Level x Cycling Pattern	20.617	3, 32	**<0.001**
	Hatch length	Level	34.054	1, 28	**<0.001**
		Cycling Pattern	11.963	3, 28	**<0.001**
		Level x Cycling Pattern	10.088	3, 28	**<0.001**
	Growth rate	Level	4.087	1, 23	0.055
		Cycling Pattern	7.393	3, 23	**0.001**
		Level x Cycling Pattern	11.359	2, 23	**<0.001**

Table S4: Spawning ripe *M. menidia* collected from Mumford Cove, CT to fertilise embryos used in static and fluctuating pCO_2 × DO experiments. Adult lengths are shown as mean total lengths (TL, cm) ± standard deviation.

Experiment	Collection date	Fertilisation date	Number of female spawners	Female lengths	Number of male spawners	Male lengths
1	05/08/2017	05/09/2017	21	9.3 ± 1.3	39	8.1 ± 1.1
2	06/08/2017	06/09/2017	28	11.0 ± 0.9	23	8.9 ± 1.2
3	14/05/2018	16/05/2018	12	9.0 ± 0.8	32	7.7 ± 1.0
4	14/06/2018	15/06/2018	22	9.6 ± 1.4	29	8.3 ± 0.8

Table S5: Seawater parameters from experiments one and two. Means ± SD are listed for pH_{NIST}, dissolved oxygen (DO), partial pressure of CO_2 (pCO_2), total alkalinity (A_T), temperature, and salinity.

Exp.	Treatment	Measured pH_{NIST}	Measured DO (mg L^{-1})	pCO_2* (µatm)	A_T (µmol kg^{-1})	Temperature (°C)	Salinity (psu)
One	Control pCO_2-Normoxic	8.18 ± 0.02	7.8 ± 0.1	370 ± 1	2001 ± 6	24.7 ± 0.4	30 ± 0
	Control pCO_2-Reduced DO	8.12 ± 0.04	4.1 ± 0.4	439 ± 2	1996 ± 7	24.6 ± 0.4	30 ± 0
	Control pCO_2-Hypoxic	8.15 ± 0.05	2.7 ± 0.4	400 ± 1	2004 ± 3	24.9 ± 0.3	30 ± 0
	Intermediate pCO_2-Normoxic	7.56 ± 0.10	7.7 ± 0.1	1826 ± 7	2005 ± 8	24.5 ± 0.3	30 ± 0
	Intermediate pCO_2-Reduced DO	7.46 ± 0.06	4.0 ± 0.4	2338 ± 5	2002 ± 4	24.7 ± 0.4	30 ± 0
	Intermediate pCO_2-Hypoxic	7.48 ± 0.08	2.7 ± 0.4	2189 ± 4	2010 ± 3	24.2 ± 0.5	30 ± 0
	Extreme pCO_2-Normoxic	7.19 ± 0.07	7.7 ± 0.1	4368 ± 19	1998 ± 9	24.0 ± 0.6	30 ± 0
	Extreme pCO_2-Reduced DO	7.22 ± 0.03	4.1 ± 0.3	4119 ± 17	2003 ± 8	24.2 ± 0.5	30 ± 0
	Extreme pCO_2-Hypoxic	7.20 ± 0.05	2.5 ± 0.3	4337 ± 36	2014 ± 17	24.3 ± 0.4	30 ± 0
Two	Control pCO_2-Normoxic	8.17 ± 0.08	7.8 ± 0.1	385 ± 2	2062 ± 11	24.4 ± 0.3	30 ± 0
	Control pCO_2-Reduced DO	8.07 ± 0.09	4.2 ± 0.3	505 ± 1	2046 ± 4	24.4 ± 0.4	30 ± 0
	Control pCO_2-Hypoxic	8.06 ± 0.09	3.1 ± 0.5	520 ± 5	2050 ± 19	24.0 ± 0.4	30 ± 0
	Intermediate pCO_2-Normoxic	7.50 ± 0.07	7.8 ± 0.1	2173 ± 22	2060 ± 21	24.7 ± 0.3	30 ± 0
	Intermediate pCO_2-Reduced DO	7.50 ± 0.05	4.1 ± 0.4	2157 ± 12	2055 ± 12	24.4 ± 0.3	30 ± 0
	Intermediate pCO_2-Hypoxic	7.50 ± 0.05	3.0 ± 0.3	2151 ± 19	2039 ± 17	24.1 ± 0.4	30 ± 0
	Extreme pCO_2-Normoxic	7.19 ± 0.11	7.7 ± 0.1	4539 ± 55	2064 ± 25	24.4 ± 0.4	30 ± 0
	Extreme pCO_2-Reduced DO	7.19 ± 0.07	4.1 ± 0.4	4512 ± 20	2060 ± 9	24.4 ± 0.4	30 ± 0
	Extreme pCO_2-Hypoxic	7.19 ± 0.06	3.0 ± 0.3	4473 ± 64	2053 ± 29	24.2 ± 0.5	30 ± 0

*pCO_2 was calculated from pH_{NIST} and A_T using CO2SYS.

Table S6 - *Seawater parameters from experiment three.* Means ± SD are listed for daily mean, minimum and maximum of pH_{NIST}, dissolved oxygen (DO) and partial pressure of CO_2 (pCO_2). Values for non-varying parameters are listed as mean ± SD over the experiment.

Level	Cycling Pattern		Measured pH_{NIST}	Measured DO (mg L^{-1})	pCO_2* (µatm)	A_T (µmol kg^{-1})	Temperature (°C)	Salinity (psu)
Control pCO_2-Normoxic	Static	Mean	8.13 ± 0.03	7.6 ± 0.2	425 ± 1	1998 ± 6	23.9 ± 0.2	30 ± 0
		Min.	-	-	-			
		Max.	-	-	-			
Intermediate pCO_2-Reduced DO	Static	Mean	7.45 ± 0.04	3.7 ± 0.5	2351 ± 6	1999 ± 5	24.6 ± 0.2	31 ± 0
		Min.	-	-	-			
		Max.	-	-	-			
	Small Diel Fluctuation	Mean	7.48 ± 0.19	3.9 ± 1.3	2147 ± 5	1983 ± 4	23.8 ± 0.2	31 ± 0
		Min.	7.13 ± 0.11	2.3 ± 0.4	1166 ± 3			
		Max.	7.73 ± 0.02	6.0 ± 0.3	4953 ± 11			
	Large Diel Fluctuation	Mean	7.48 ± 0.35	3.8 ± 1.4	2163 ± 11	2004 ± 23	24.0 ± 0.2	31 ± 1
		Min.	6.84 ± 0.06	1.4 ± 0.3	521 ± 2			
		Max.	8.05 ± 0.02	6.2 ± 0.2	9926 ± 59			
	Tidal Fluctuation	Mean	7.44 ± 0.32	4.3 ± 1.3	2426 ± 42	2015 ± 22	23.9 ± 0.2	31 ± 1
		Min.	6.90 ± 0.07	1.6 ± 0.3	699 ± 13			
		Max.	7.94 ± 0.02	6.1 ± 0.4	8667 ± 142			
Extreme pCO_2-Hypoxic	Static	Mean	7.18 ± 0.07	2.4 ± 0.6	4447 ± 39	1988 ± 5	24.1 ± 0.2	31 ± 1
		Min.	-	-	-			
		Max.	-	-	-			
	Small Diel Fluctuation	Mean	7.26 ± 0.20	3.1 ± 0.8	3705 ± 39	1983 ± 9	23.9 ± 0.2	31 ± 1
		Min.	6.85 ± 0.08	1.9 ± 0.2	1872 ± 21			
		Max.	7.54 ± 0.03	4.2 ± 0.2	9590 ± 97			
	Large Diel Fluctuation	Mean	7.18 ± 0.33	3.3 ± 1.6	4409 ± 13	1986 ± 6	24.0 ± 0.2	31 ± 0
		Min.	6.63 ± 0.07	1.1 ± 0.2	1058 ± 3			
		Max.	7.77 ± 0.02	5.3 ± 0.2	15970 ± 48			
	Tidal Fluctuation	Mean	7.12 ± 0.32	3.4 ± 1.1	5177 ± 129	2008 ± 38	24.2 ± 0.2	31 ± 1
		Min.	6.61 ± 0.07	1.6 ± 0.5	1349 ± 36			
		Max.	7.68 ± 0.09	5.0 ± 0.5	17013 ± 417			

*pCO_2 was calculated from pH_{NIST} and A_T using CO2SYS.

Table S7 - *Seawater parameters from experiment four.* Means ± SD are listed for daily mean, minimum and maximum of pH_{NIST}, dissolved oxygen (DO) and partial pressure of CO_2 (pCO_2). Values for non-varying parameters are listed as mean ± SD over the experiment.

Level	Cycling Pattern		Measured pH_{NIST}	Measured DO (mg L^{-1})	pCO_2* (µatm)	A_T (µmol kg^{-1})	Temperature (°C)	Salinity (psu)
Control pCO_2-Normoxic	Static	Mean	8.17 ± 0.07	7.5 ± 0.2	379 ± 2	2025 ± 10	23.7 ± 0.4	32 ± 1
		Min.	-	-	-			
		Max.	-	-	-			
Intermediate pCO_2-Reduced DO	Static	Mean	7.45 ± 0.04	4.2 ± 0.4	2337 ± 19	2032 ± 5	23.8 ± 0.5	32 ± 1
		Min.	-	-	-			
		Max.	-	-	-			
	Small Diel Fluctuation	Mean	7.53 ± 0.17	5.7 ± 0.7	1931 ± 2	2020 ± 3	23.5 ± 0.5	31 ± 0
		Min.	7.34 ± 0.09	4.0 ± 0.6	876 ± 1			
		Max.	7.85 ± 0.04	6.6 ± 0.5	3059 ± 5			
	Large Diel Fluctuation	Mean	7.49 ± 0.31	5.5 ± 0.9	2119 ± 2	2030 ± 2	23.6 ± 0.4	32 ± 0
		Min.	6.89 ± 0.11	3.0 ± 0.2	747 ± 1			
		Max.	7.91 ± 0.03	6.1 ± 0.3	8810 ± 8			
	Tidal Fluctuation	Mean	7.40 ± 0.27	5.4 ± 0.8	2672 ± 3	2024 ± 2	23.6 ± 0.6	31 ± 0
		Min.	6.87 ± 0.04	3.2 ± 0.4	948 ± 1			
		Max.	7.82 ± 0.06	6.6 ± 0.3	9277 ± 10			
Extreme pCO_2-Hypoxic	Static	Mean	7.18 ± 0.05	3.3 ± 0.2	4489 ± 6	2028 ± 3	23.6 ± 0.5	31 ± 0
		Min.	-	-	-			
		Max.	-	-	-			
	Small Diel Fluctuation	Mean	7.25 ± 0.14	4.3 ± 0.5	3783 ± 7	2016 ± 7	23.7 ± 0.6	32 ± 1
		Min.	7.02 ± 0.06	3.0 ± 0.2	2341 ± 5			
		Max.	7.45 ± 0.03	5.2 ± 0.3	6490 ± 11			
	Large Diel Fluctuation	Mean	7.29 ± 0.27	4.4 ± 1.2	3478 ± 24	2027 ± 1	23.7 ± 0.5	31 ± 1
		Min.	6.74 ± 0.04	2.6 ± 0.3	1258 ± 10			
		Max.	7.71 ± 0.03	5.7 ± 0.2	12624 ± 79			
	Tidal Fluctuation	Mean	7.18 ± 0.25	4.5 ± 1.0	4425 ± 30	2010 ± 13	23.9 ± 0.5	31 ± 0
		Min.	6.77 ± 0.04	2.7 ± 0.3	1515 ± 10			
		Max.	7.63 ± 0.04	5.9 ± 0.3	11658 ± 78			

*pCO_2 was calculated from pH_{NIST} and A_T using CO2SYS.
